# New structure of step-up DC-DC converter based on three winding coupled inductor with high gain capability featuring integrated renewable energy applications

**DOI:** 10.1038/s41598-024-83502-4

**Published:** 2024-12-30

**Authors:** Pouneh Aghakhanlou, Fatemeh Falahi, Ali Nadermohammadi, Hasan Sarikhan, Seyed Hossein Hosseini, Naghi Rostami, Mehran Sabahi

**Affiliations:** 1 Faculty of Electrical and Computer Engineering, University of Tabriz, Tabriz, 51666-16471 Iran; 2 Engineering Faculty, Near East University, Nicosia, 99138 Turkey

**Keywords:** Electrical and electronic engineering, Renewable energy

## Abstract

This research presents an innovative design for a non-isolated DC-DC converter, which utilizes a single switch in a high step-up configuration. The key element of this design is a three-winding coupled inductor (TWCI), which plays a crucial role in achieving a substantial voltage increase. By utilizing a low duty cycle, the converter minimizes conduction losses in the power switch, resulting in enhanced efficiency. The converter offers several benefits, including reduced voltage stress on both power switches and diodes, a common ground, high efficiency, a simple structure and control mechanism, and fewer components. The study thoroughly examines the converter’s operational modes and provides a comparison with other types of converters to highlight its distinctive characteristics. To validate the converter’s performance, a 400 W prototype was developed and tested in the lab, operating with a 20 V input, a 260 V output, and a switching frequency of 50 kHz.

## Introduction

**Fig. 1:**
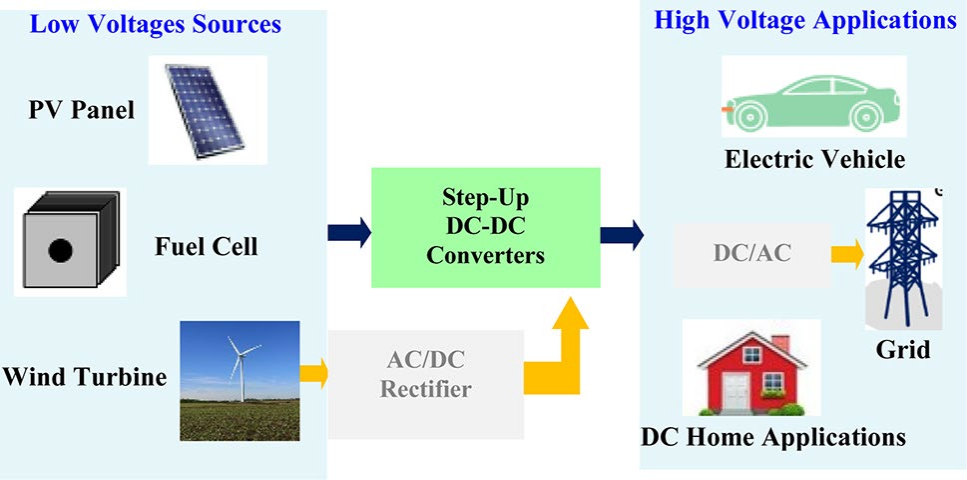
Energy flow from low-voltage sources to high-voltage applications.

Over the past decade, there has been a significant focus on reducing carbon emissions and promoting the widespread use of renewable energy sources. Based on Fig. 1, these energy sources are considered the leading alternatives to fossil fuel. Photovoltaic (PV) energy especially, has gained significant popularity because of its wide availability, sustainability, and minimal environmental impact. However, the DC voltage produced by PV panels and fuel cells is typically low and unsuitable for direct grid integration. Consequently, high voltage DC-DC boost converters play a fundamental role in renewable energy applications by addressing the need to boost the relatively low voltage output from renewable energy sources to a higher voltage suitable for use of further processing. These converters are essential for converting electricity from renewable sources, aiding in the fight against air pollution, environmental harm, and global warming caused by fossil fuels. Lately, numerous step-up DC-DC converters employing various voltage boosting techniques have been introduced in^1–3^. DC-DC converters often use different voltage-boosting techniques, such as coupled inductors, switched capacitors, multiplier cells, and high-frequency transformers, to achieve higher voltage outputs. In isolated converters, achieving a high voltage conversion ratio is accomplished through the use of transformers with significantly high turn ratios. Nevertheless, excessively large turn ratios result in significant leakage inductance, which diminishes converter efficiency and raises the voltage stress on switches^4,5^. In recent years, the coupled-inductor (CI) technique, which recaptures energy from leakage inductance, has been employed to improve the voltage conversion ratio. This has led to the development of numerous high voltage boost converters based on CI, offering advantages such as reduced voltage stress, increased voltage gain, and enhanced efficiency. The presence of secondary leakage inductance in these converters effectively reduces reverse recovery ringing in the output diode, offering improved performance. However, it’s important to note that achieving higher voltage gain through increased turn ratios and extremely high duty cycles can result in greater EMI noise and power losses. A novel configuration that integrates a coupled- inductor and capacitors to achieve the desired voltage gain is presented. The voltage gain is greatly improved by using a coupled inductor to charge a capacitor, which naturally limits the voltage across the switch^6,7^. Cascaded inductor techniques can also provide high voltage gain while maintaining low voltage stress on the power switch^8,9^. However, these approaches necessitate a substantial number of components. References^10–13^, use the voltage multiplier method to achieve high voltage gains. However, this method requires a large number of components, which increases both the cost and size of the converter. To address these issues while enhancing the voltage conversion ratio, reference^14^ proposes a structure using an active switched inductor (ASL) along with a trans-inverse coupled inductor technique, this reduces the number of components. In references^15^ and^16^, high step-up DC-DC converters that feature minimal input current ripple and a high voltage ratio were introduced. Conversely, the proposed design includes a considerable number of components, which contributes to an increase in overall cost. In^17^, a high-gain, two-phase interleaved DC-DC converter with a coupled inductor is developed to step up low input voltage sources within a distributed generation system (DGS). This converter can handle a wide range of load variations when integrated to DGS. Moreover^18^, , presents an interleaved high step-up DC-DC converter with an integrated transformer. This design effectively eliminates leakage inductance and resolves the reverse recovery issue. In^19^, a dual-output high voltage DC-DC converter is introduced, offering low input current ripple and reduced voltage stress on semiconductor components. Structure in^20^, features a two output DC-DC converter with a high voltage conversion rate, utilizing coupled inductors and voltage multiplier cells (VMCs) for renewable applications is presented. This configuration includes two separate output ports, each providing distinct voltage gains. References^21^ and^22^, describe a quadratic base design that overcomes the voltage limitations of conventional quadratic bases, utilizing CI to boost gain. In reference^23^, a high step-up DC-DC converter is introduced, utilizing a combination of the coupled-inductor approach and a voltage multiplier cell technique. Another non isolated converter based on CI with low input current ripple which is suitable for renewable energies is presented in^24^. Switched-cell boost converters can be categorized into switched-inductor and switched-capacitor boost converters, depending on the type of energy storage element employed. In CIs, the voltage stress on the power devices relatively high, whereas in SC-C, the current through the power switch is high, both significantly impacting efficiency. More recently, several high step-up DC-DC converters incorporating three-winding CIs have been proposed^25–30^. The idea of expanding the ASL units was improved performance by sustaining the converter inductors with PSI cells in^31,32^. These designs provide greater flexibility in controlling voltage stress and voltage gain; however, the resulting voltage gain is still limited and could benefit from further enhancement. Incorporating a coupled inductor eliminates the requirement for operating the power switch at a high duty cycle, leading to enhanced efficiency. Moreover, the reduced voltage stresses make it possible to employ semiconductors with lower voltage ratings. These studies^33–35^, introduce DC-DC converters engineered for significant voltage boosting, utilizing a coupled inductor with two windings and multiplier cells. This design strategy seeks to achieve high voltage gain with a low duty cycle, which in turn reduces conduction losses in the power switch and improves overall efficiency. This study presents a non-isolated DC-DC converter that achieves a significant voltage boost using a three-winding coupled inductor and a single power switch. The design effectively reaches a high voltage conversion ratio while keeping voltage stress on both the switch and diodes low, even in scenarios with elevated output voltages. The voltage stress on the single switch is kept low, making it possible to use a component with a lower voltage rating switch on resistance to reduce conduction loss, thereby enhancing efficiency. The proposed topology offers numerous benefits, including an, elevated voltage conversion ratio at low cycle durations achieved with a low winding ratio of the coupled inductor, causing decreased leakage inductance. Operating at low duty cycles and under zero-current switch conditions also mitigates the reverse recovery problem of diodes. However, this topology achieves high voltage gains independent of the duty cycle, enabling it to be adjusted anywhere from 0 to 1. Additionally, it employs only one power switch, and can produce a high output voltage with small duty cycle, which contributes to low conduction losses and reduced voltage stress on semiconductor components. Furthermore, it effectively recovers energy from leakage inductance and maintains high efficiency. The paper substantiates the proposed topology’s performance by offering insights into operation principles, conducting steady- state and efficiency analysis. The results of the comparison with other converters are then presented. To validate the performance of the proposed converter and confirm the mathematical relationships and theoretical analysis, a 400-W prototype was built in the lab. This prototype operates with an input voltage of 20 V, an output voltage of 260 V, and a switching frequency of 50 kHz.

## Proposed converter and operation modes

**Fig. 2:**
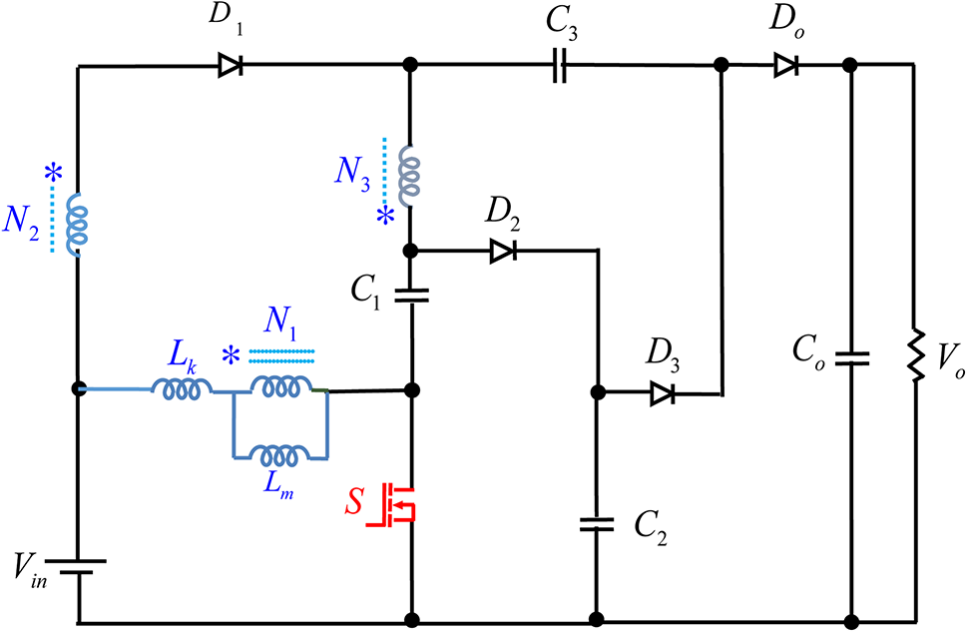
Power circuitry of the suggested design.

**Fig. 3:**
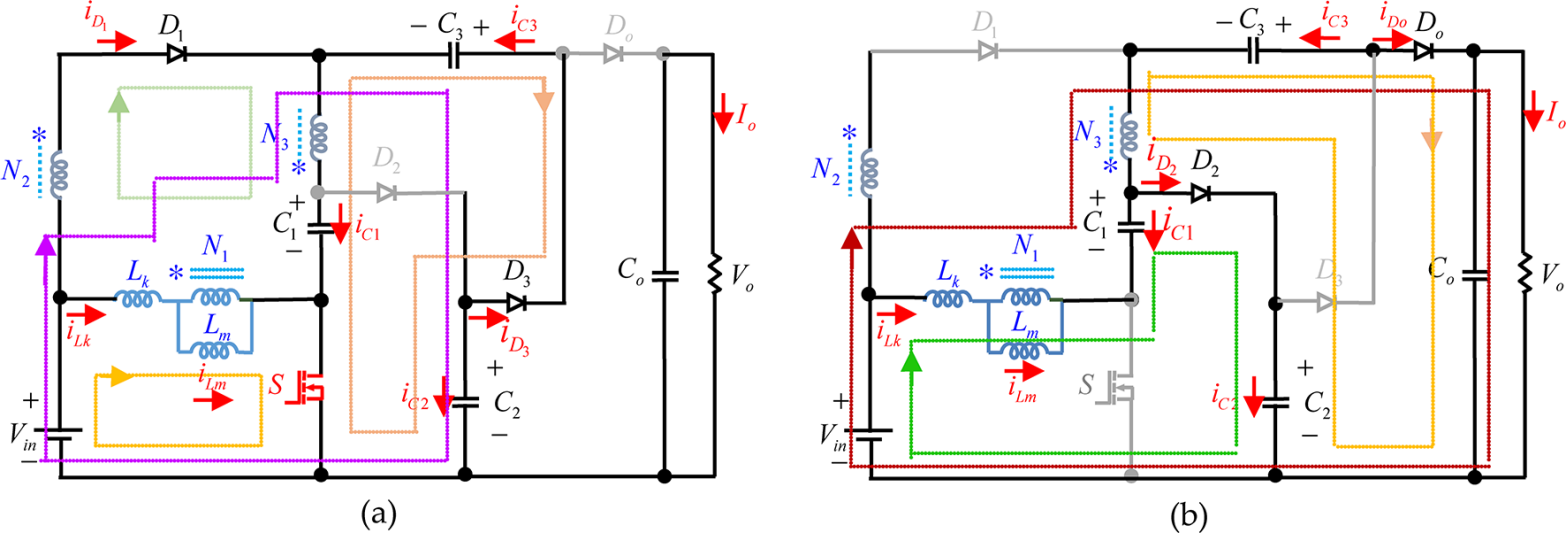
Equivalent circuit models of the suggested design (**a**) First switching interval, (**b**) Second switching interval.

**Fig. 4:**
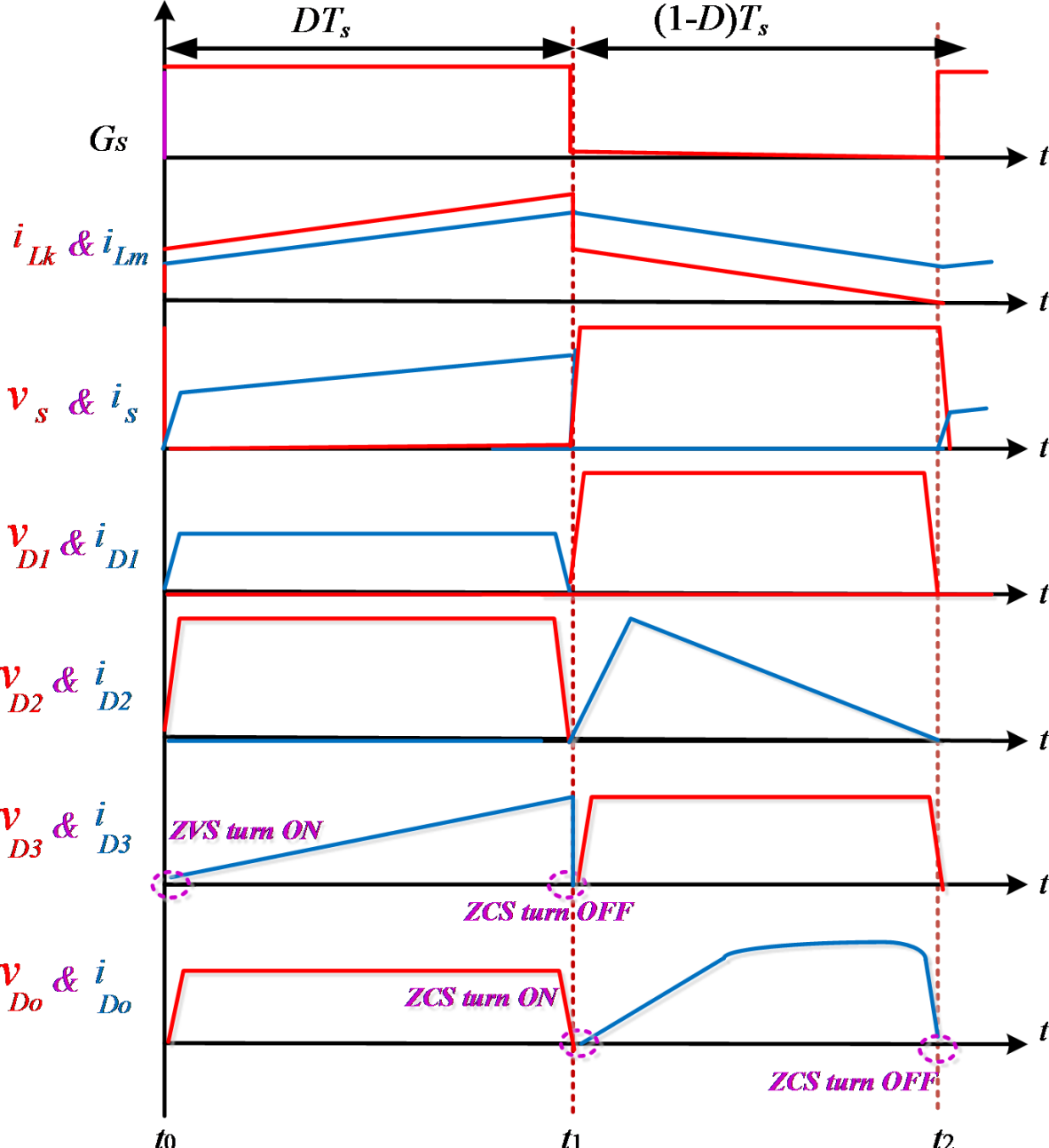
Main waveforms of the proposed topology.

The design presented in this paper features a non-isolated DC-DC converter that employs a single switch along with a coupled inductor. This configuration is illustrated in Fig. 2. The proposed structure consists of single switch $S$, four diodes ${D_1}$, ${D_2}$, ${D_3}$ and ${D_o}$, four capacitors ${C_1}$, ${C_2}$ and ${C_3}$, an output filter capacitor ${C_o}$, and a TWCI comprises N_1_ (number of primary side winding), N_2_ (number of secondary side winding) and N_3_ (number of tertiary side winding). The coupling coefficient is calculated as k = L_m_ / (L_m_ + L_k_), where L_m_ represents the mutual inductance and *L*_*k*_ denotes the leakage inductance. The evaluation of the proposed converter is carried out under continuous conduction mode (CCM), where a direct current (DC) input voltage source is assumed to be free of any ripple. Furthermore, the output filter capacitor ensured to be sufficiency large, and switch and diodes are assumed to be ideal. As previously stated, all semiconductor components are assumed to be ideal, meaning that diode conduction losses and the on-state resistance of the active switch are neglected. Additionally, the effects of the equivalent series resistance of passive components such as capacitors and inductors are also disregarded. Furthermore, the capacitors are assumed to have a high capacitance, ensuring that their voltage remains constant throughout operation. Subsequently, the high step-up topology outlined in this work consists of two switching subintervals, as demonstrated in Fig. 3. Moreover, Fig. 4 showcases the essential voltage and current waveforms related to this proposed configuration.

**First switching interval [Fig.** 3**(a),** ***t***_**0**_ − ***t***_**1**_**]**: At the initiation of this mode, the power switch *s* begin conducting simultaneously, and diodes D_1_ and D_3_ are forward biased, initiating the conduction of D_3_ under zero voltage switching (ZVS) condition, while the diodes D_2_, and D_o_ become reverse biased. The input voltage source V_in_ energizes the magnetizing inductor L_m_, causing the current i_Lm_ to rise steadily over time. Simultaneously, capacitor *C*_*1*_ is charged through a circuit pathway consisting of the tertiary winding of the CI, diode D_3_, and capacitor C_3_. The equivalent circuit is illustrated in Fig. 3(a), with its corresponding key waveform depicted in Fig. 4. The derivation of the associated equations is presented below

1 $${V_1}={V_{Lm}}+{V_{Lk}}=\frac{{{V_{Lm}}}}{k}$$

2 $$- {V_{in}} - {V_{N2}} - {V_{N3}}+{V_{C1}}=0$$

3 $$- {V_{in}}+{V_1} - {V_{C1}}+{V_{N3}} - {V_{C3}}+{V_{C2}}=0$$

4 $$- {V_{N2}} - {V_{N3}}+{V_{C1}} - {V_1}=0$$

5 $$- {V_{C1}}+{V_{N3}} - {V_{C3}}+{V_{C2}}=0$$

6 $${V_{Lm}}=k.{V_{in}}$$

**Second switching interval [Fig.** 3**(b),** ***t***_**1**_ − ***t***_**2**_**]**: During the second operation, When the power switch S, is turned off and diodes of D_2_ and D_o_ become forward-biased. Under zero-current switching (ZCS) conditions, diode D_o_ starts conducting, while all the remaining diodes are in a reverse-biased state. Consequently, the magnetizing inductor L_m_ discharges, transferring energy to capacitor *C*_*2*_ through the circuit that includes L_m_, diode D_2_, capacitor C_2_, and the input voltage source V_in_. Thus, the magnetizing inductor L_m_ reaches to its minimum value linearly and the capacitor C_2_ is fully charged at the end of this mode. The capacitor C_1_ discharged through the L_m_, D_2_, and *C*_*2*_. At the end of this subinterval, the diode D_2_ is turned-off at the ZCS condition. Based on Kirchhoff’s Voltage Law (KVL), the voltage relationships for this operational mode can be expressed as follows.

7 $$- {V_{in}}+{V_1}+{V_{C2}} - {V_{C1}}=0$$

8 $${V_{N3}}=n{V_{Lm}}$$

9 $$- {V_{C2}}+{V_{N3}} - {V_{C3}}+Vo=0$$

10 $$- {V_{in}}+{V_1} - {V_{C1}}+{V_{N3}} - {V_{C3}}+{V_o}=0$$

11 $${V_{Lm}}=\frac{{D.k.{V_{in}}}}{{D - 1}}$$

### Voltage gain calculation

In this part of the study, we assess the voltages across the capacitors and calculate the voltage gain of the proposed converter using the volt-second balance principle applied to the inductors across a specified time interval. Figure 6 illustrates the three-dimensional representation of the voltage gain.

The following conclusions can be drawn by utilizing the volt-second balance principle:

12 $$\int\limits_{0}^{{{T_s}}} {{V_{Lm}}} dt=0$$

By inserting Eqs. (1) and (7) into Eq. (12), the resulting voltage across capacitor *C*_*1*_ is determined:

13 $${V_{C1}}={V_{in}}\left( {2kn+1} \right)$$

The voltage of capacitor *C*_*2*_ derived from Eqs. (5), (8), and (13).

14 $${V_{C2}}=\frac{{{V_{in}}\left( {2kn - 2Dkn - D+2} \right)}}{{1 - D}}$$

The voltage of capacitor *C*_*3*_ can be calculated as:

15 $${V_{C3}}=\frac{{{V_{in}}\left( {kn - Dkn+1} \right)}}{{1 - D}}$$

And finally, the output voltage to input voltage ratio by gusting the relations (5), (6), (11), (12) and (13) the voltage gain (M), is obtained as follow:

16 $$M=\frac{{{V_o}}}{{{V_{in}}}}=\frac{{3kn - 2Dkn - D+3}}{{1 - D}}$$

**Fig. 5:**
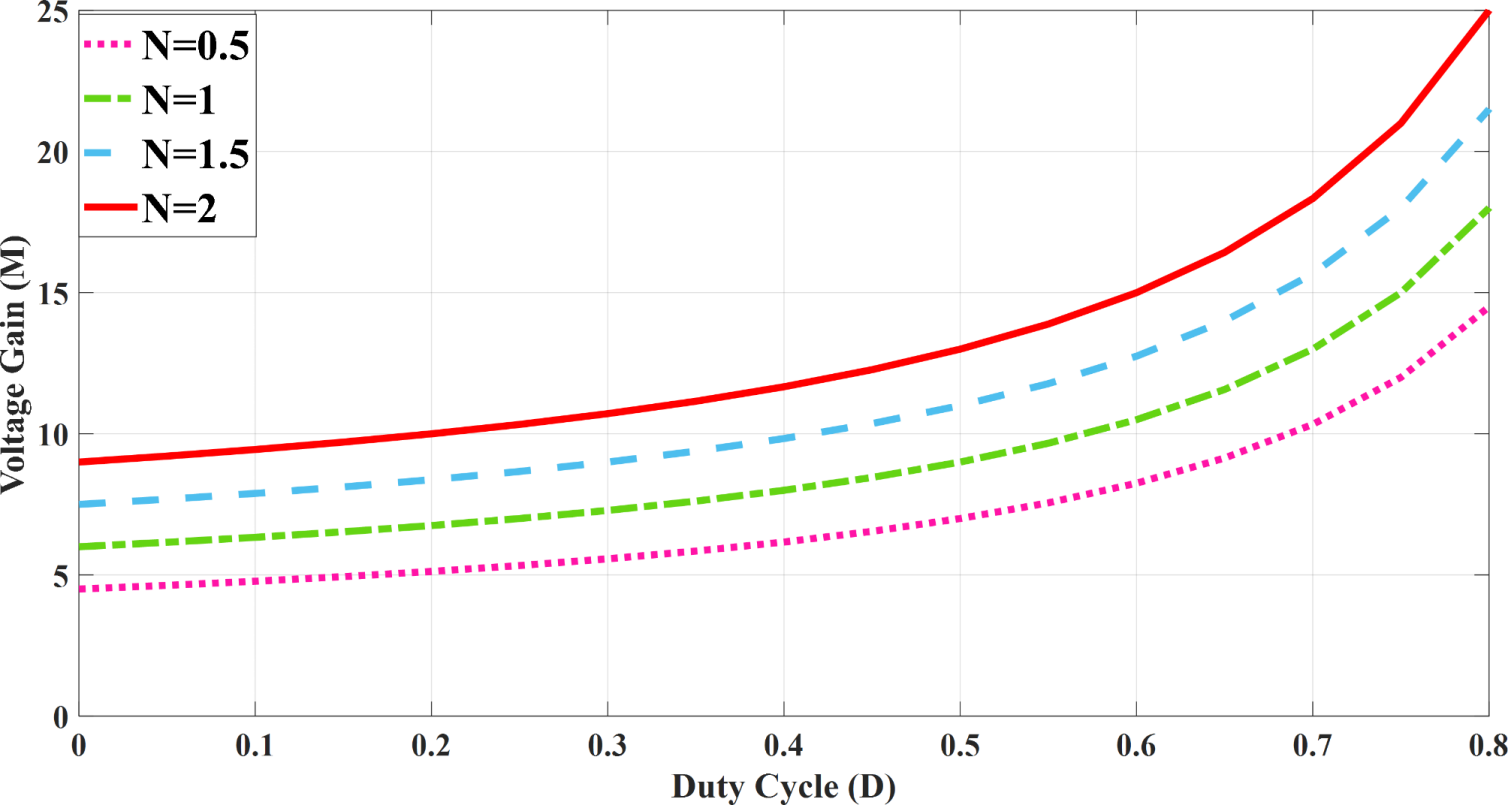
The voltage gain for versus duty cycle and different values of turn ratio (for k = 1).

Figure 5 illustrates the correlation between the voltage gain and duty cycle for different turn ratios. It is evident that as the duty cycle increases across a broader range, the voltage gain rises accordingly, reflecting the changes in turns ratio. Therefore, the voltage gain of the proposed topology is drawn for different turn ratios from 0.5 to 2 and 0 < D < 1.

**Fig. 6:**
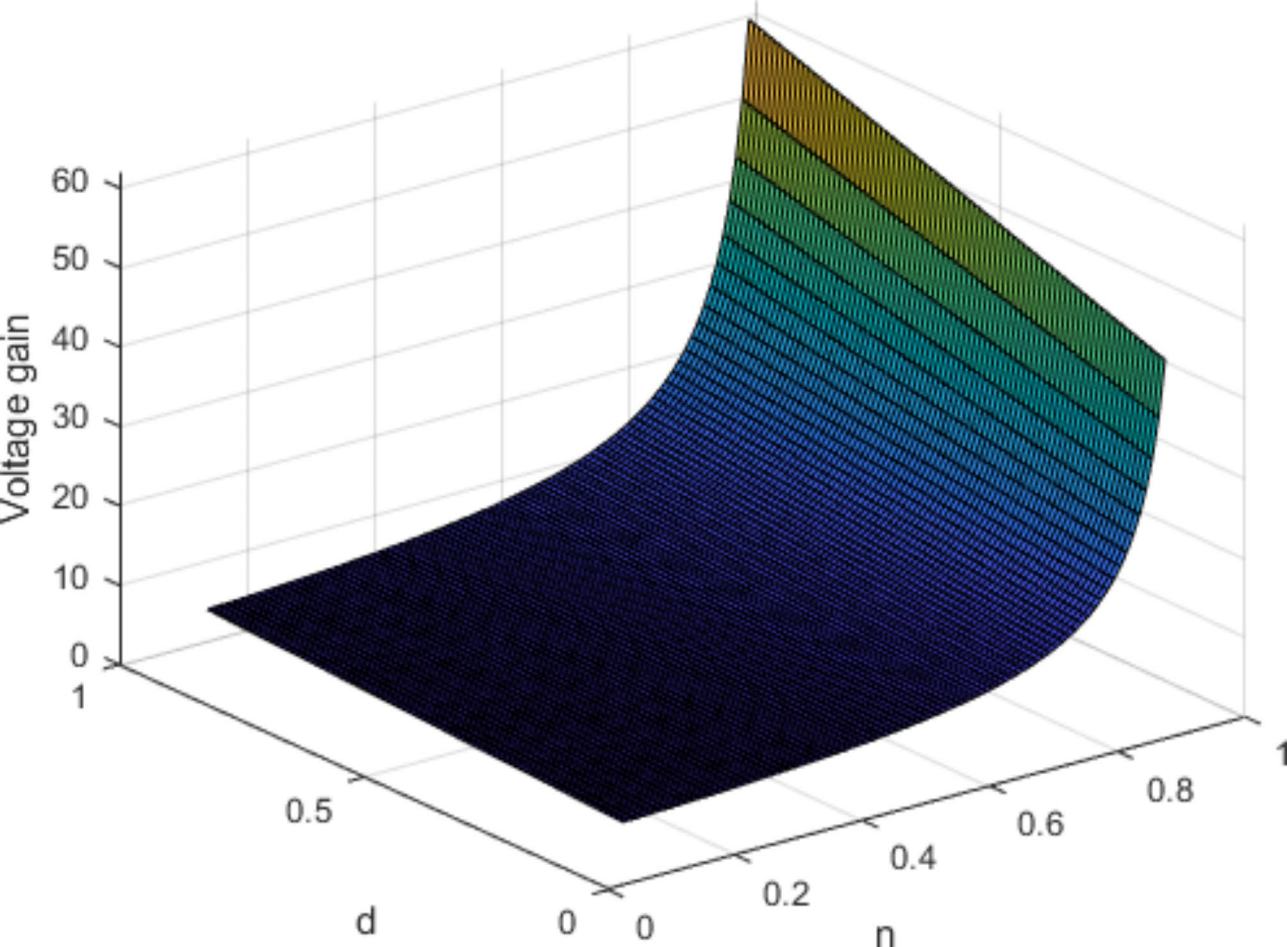
The 3-D curve voltage gain versus duty cycle and turn ratio (for k = 1).

## Voltage stresses of semiconductors

The voltage stresses experienced by the single switch and diodes are evaluated by examining the behavior of the proposed converter across different operational modes throughout a complete switching cycle. The voltage stress on the single switch is specifically derived based on the analysis of the first operating mode, as illustrated in Fig. 11:

17 $${V_s}=\frac{{{V_{in}}}}{{1 - D}}$$

18 $${V_{D1}}=\frac{{{V_{in}}(2kn+1)}}{{1 - D}}$$

19 $${V_{D2}}=\frac{{{V_{in}}}}{{1 - D}}$$

20 $${V_{D3}}={V_{Do}}=\frac{{{V_{in}}(kn+1)}}{{1 - D}}$$

21 $${V_{Do}}=\frac{{ - {V_i}}}{{{{(1 - D)}^2}}}=\frac{{ - (1+N)}}{{(2+N(3+N))}}{V_o}$$

### Average currents of semiconductors

Following Fig. 3 and the relationships of the operation modes, the maximum currents of the semiconductor devices are presented as follows (Table 1):

22 $${I_{S(peak)}}=\frac{{(M - 1)}}{D}{I_o}$$

23 $${I_{D1(peak)}}={I_{D3(peak)}} \approx \frac{{2{I_o}}}{D}$$

24 $${I_{D2(peak)}}={I_{Do(peak)}} \approx \frac{{2{I_o}}}{{1 - D}}$$

**Table 1:** The average and RMS values of the components.

Components		Average	Rms
Switch	$S$	${I_{S(ave)}}=(M - 1){I_o}$	${I_{S(rms)}}=\frac{{(M - 1)}}{{\sqrt D }}{I_o}$
Diodes	${D_1}$	${I_{D1(ave)}}={I_o}$	${I_{D1(rms)}}=2{I_o}\sqrt D$
	${D_2}$	${I_{D2(ave)}} \approx {I_o}$	${I_{D2(rms)}}=2{I_o}\sqrt {\frac{1}{{1 - D}}}$
	${D_3}$	${I_{D3(ave)}}={I_o}$	${I_{D3(rms)}}=2{I_o}\sqrt D$
	${D_o}$	${I_{Do(ave)}}={I_o}$	${I_{Do(rms)}}=2{I_o}\sqrt {\frac{1}{{(1 - D)}}}$
Capacitors	${C_1}$	${I_{C1(rms)}}=\frac{{2{I_o}}}{{\sqrt D }}+\frac{{2{I_o}}}{{\sqrt {(1 - D)} }}$	
	${C_2}$	${I_{C2(rms)}}=\frac{{2{I_o}}}{{\sqrt D }}+\frac{{2{I_o}}}{{\sqrt {(1 - D)} }}$	
	${C_3}$	${I_{C3(rms)}}=\frac{{2{I_o}}}{{\sqrt D }}+\frac{{2{I_o}}}{{\sqrt {(1 - D)} }}$	
Inductors	${L_k}$	${I_{Lk(ave)}}=(1 - D)M{I_o}$	
	${L_m}$	${I_{Lm(ave)}} \approx \frac{M}{2}{I_o}$	

### Efficiency calculation

**Fig. 7:**
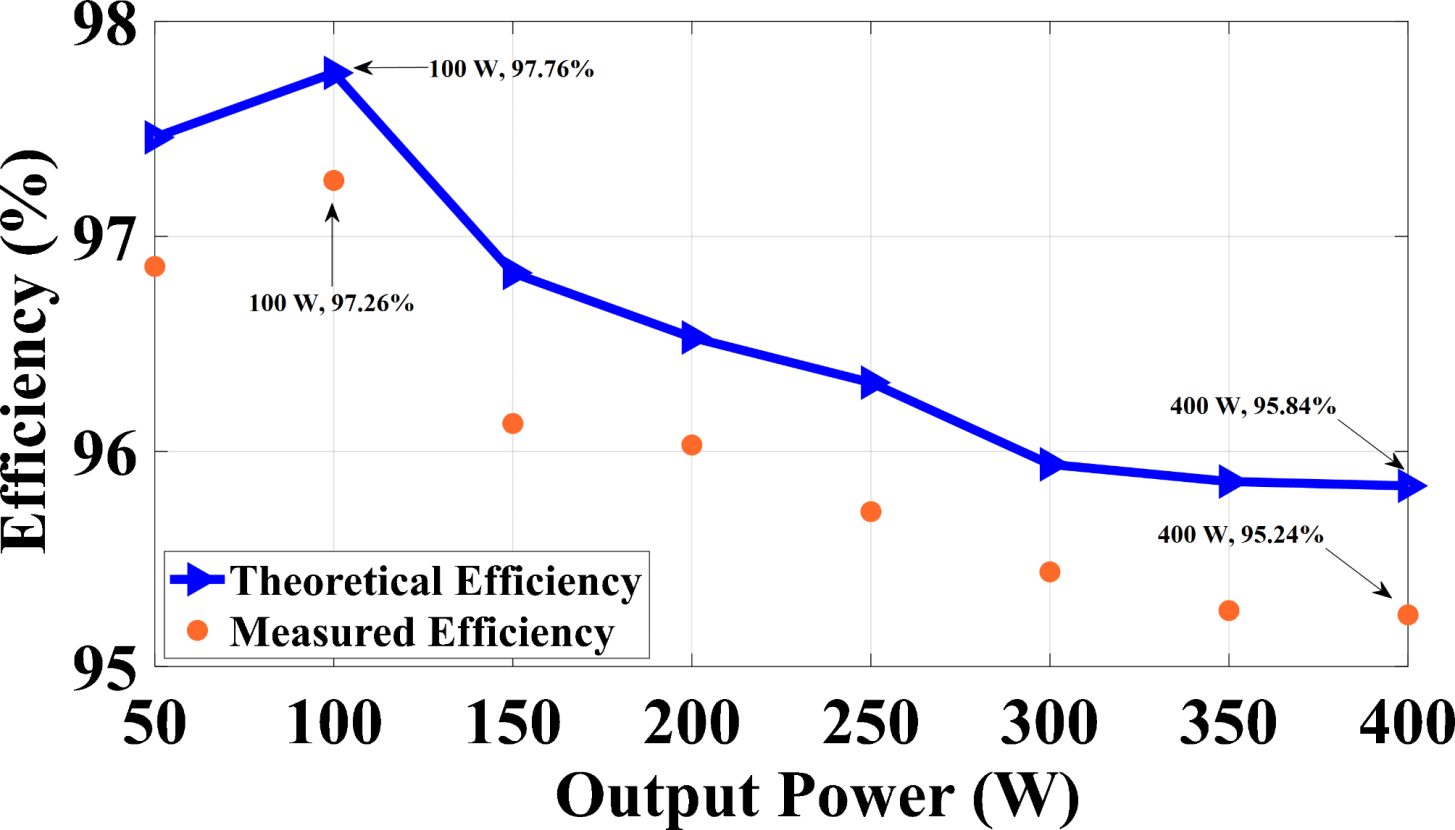
The anticipated efficiency of the suggested high step-up design in relation to output power.

**Fig. 8:**
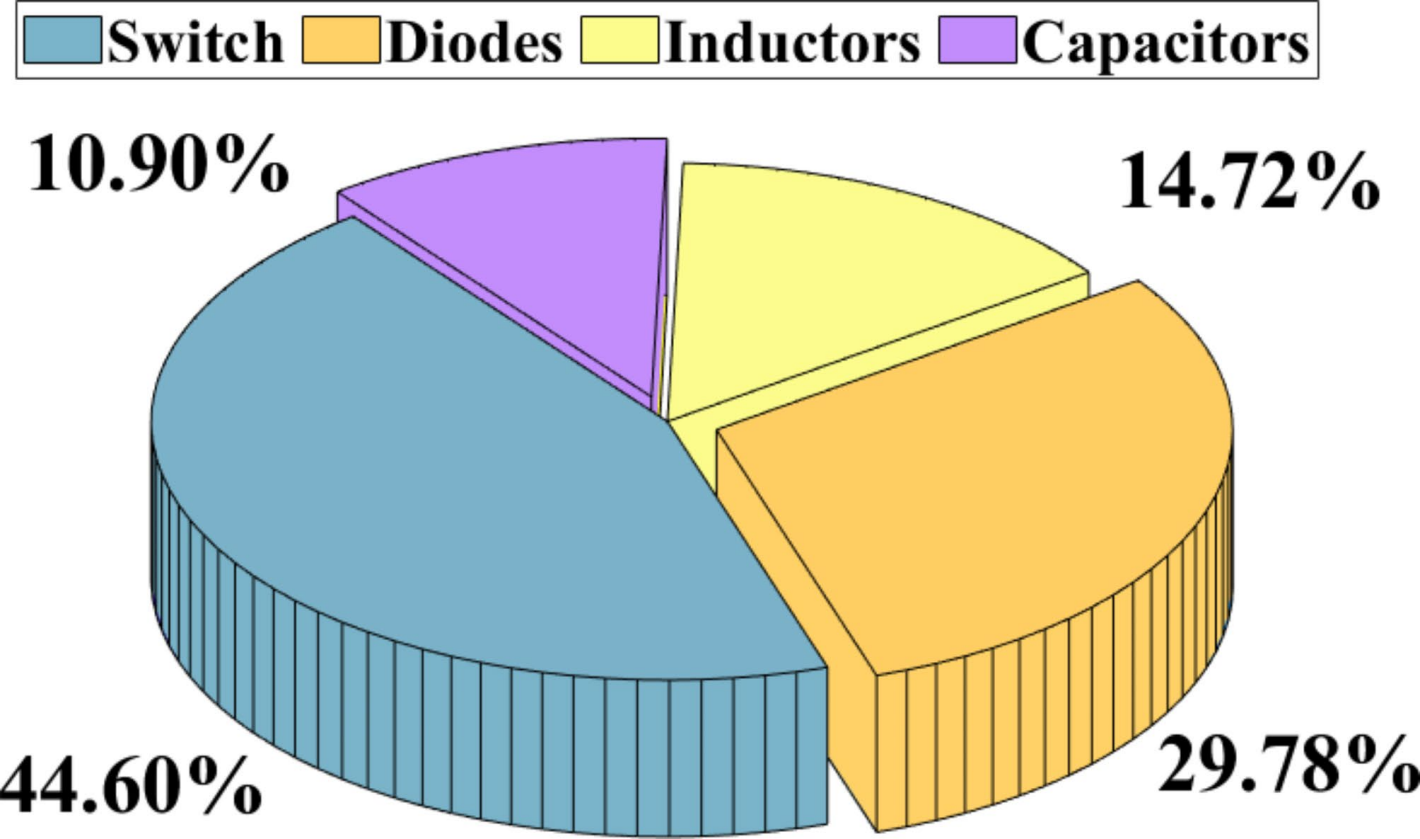
Assessed power loss figures for the components (P_Out_= 400 W, V_in_= 20 V and Vo = 260 V).

By referencing equations (25) to (31), the analytical efficiency of the proposed circuit can be derived, and the theoretical and experimental efficiencies of the proffered circuit versus output power are graphed in Fig. 7. In practical scenarios, parasitic elements within circuit components lead to conduction power losses, which in turn reduce the efficiency of the converter. To evaluate the efficiency of the proposed structure, these parasitic elements mist be taken into account. The relevant parasitic components are outlined in Table 6, and a simplified circuit diagram is presented in Fig. 8 to facilitate the analysis of conduction losses. This section is dedicated to evaluating the conduction and switching losses of the proposed design, aiming to accurately assess its overall efficiency. To do this, key parameters are identified, including the internal resistors of diodes (r_D_), power switch (*r*_*S*_), inductor (r_Lm_), capacitors (r_C_), forward drop voltage of diodes (V_FD_), and forward drop voltage of switch (V_F_). By utilizing these values, the conduction and switching losses associated with the power switch and diodes are calculated as outlined below:

25 $${P_S}={V_{DS}}{I_{S(ave)}}({t_{on}}+{t_{off}})/2{T_S}+{r_{DS,on}}{I_{S(rms)}}^{2}$$

The total power losses of the diodes can be calculated using the previously mentioned equations.

26 $${P_D}={V_{FD}}{I_{D(ave)}}+{I_{D(rms)}}^{2}{r_D}$$

Additionally, the power losses associated with the capacitors are determined in the following manner:

27 $${P_C}={r_{Ci}}{I_{C(rms)}}^{2}$$

Moreover, the magnetic power losses can be calculated as:

28 $${P_{MC}}={r_L}{I_{L(rms)}}^{2}+{P_{Core({L_k},T)}}$$

The total power losses of the proposed topology can be computed as outlined below:

29 $${P_{Loss(T)}}={P_S}+{P_D}+{P_C}+{P_{MC}}$$

The efficiency of the suggested converter (*η*) is calculated as:

30 $$\eta =\frac{{{P_{out}}}}{{{P_{out}}+{P_{Loss}}}}$$

In this context, P_out_ signifies the output power of the proposed converter and is expressed as:

31 $${P_{out}}=\frac{{{V_o}^{2}}}{{{R_o}}}$$

## Key parameter design guidance

### Capacitors design

The design of the capacitors takes into account the average current flowing through them during each switching interval, along with their voltage levels, the duty cycle, the permissible fluctuation range (*x*_*c*_*%*), and the switching frequency. Based on these factors, the minimum required capacitance values for C_1_ through C_O_ can be calculated as follows:

32 $${C_1} \geqslant \frac{{2{I_O}}}{{{f_s} \times {V_{in}} \times (2kn+1) \times {x_{C1}}\% }}$$

33 $${C_2} \geqslant \frac{{{I_O} \times (1 - D)}}{{{f_s} \times {V_{in}} \times (2kn - 2Dkn - D+2) \times {x_{C2}}\% }}$$

34 $${C_3} \geqslant \frac{{{I_O} \times (1 - D)}}{{{f_s} \times {V_{in}} \times (kn - Dkn+1) \times {x_{C3}}\% }}$$

35 $${C_o} \geqslant \frac{{{I_O} \times (1 - D)}}{{{f_s} \times {V_{in}} \times (3kn - 2Dkn - D+3) \times {x_{Co}}\% }}$$

### Coupled inductor design

When designing the magnetizing inductance L_m_, it is assumed that the average current flowing through the inductance exceeds half of the amplitude of its current ripple ($2{I_{L(ave)}} \geqslant \Delta {i_L}$). This assumption ensures that the circuit operates in continuous conduction mode. By utilizing the determined standing voltages and average currents, the minimum required value for L_m_ is given as follows:

36 $${L_m} \geqslant \frac{{{V_{in}} \times D \times (1 - D)}}{{{f_s} \times M{\mkern 1mu} \left( {1 - {\text{Io}}} \right) \times {x_{Lm}}\% }}$$

## Small-signal modeling

In this analysis, we consider all power semiconductors, the coupled inductor, and capacitors as ideal components. The coupled inductor features parasitic series resistances, denoted as r_L_, while the capacitors exhibit their own parasitic series resistances, referred to as r_C_. By employing the state-space averaging technique, we develop both the average model and the small-signal model for our analysis. The system equations for each operational mode are derived and averaged over the commutation period, considering the time duration of each mode. For both operational modes, the system equations are represented as follows:

37 $$\left[ {\begin{array}{*{20}{c}} {{{\dot {I}}_{Lm}}} \\ {{{\dot {V}}_{C1}}} \\ {{{\dot {V}}_{C2}}} \\ {{{\dot {V}}_{C3}}} \\ {{{\dot {V}}_{CO}}} \end{array}} \right]=[{A_j}]\left[ {\begin{array}{*{20}{c}} {{I_{Lm}}} \\ {{V_{C1}}} \\ {{V_{C2}}} \\ {{V_{C3}}} \\ {{V_{CO}}} \end{array}} \right]+[{B_j}]{V_{in}}$$

Where j = 1, 2.

The control approach for the proposed converter utilizes the pole placement technique, grounded in a small-signal model that is derived from the state-space averaging method. In this approach, the small-signal modeling technique distinguishes between state variables and control inputs, dividing them into two separate components: fixed ($\bar {X},\bar {D}$) and variable ($\tilde {x},\tilde {d}$).

38 $$\left\{ \begin{gathered} X=\bar {X}+\tilde {x} \hfill \\ D=\bar {D}+\tilde {d} \hfill \\ \end{gathered} \right.$$

Applying this method to the state-space averaged model, and omitting the squared terms, enables the derivation of the small-signal model for the proposed design:

39 $$\left\{ \begin{gathered} \dot {\tilde {x}}=A\tilde {x}+B\tilde {u} \hfill \\ y=C\tilde {x}+D\tilde {u} \hfill \\ \end{gathered} \right.$$

Where variable states ($\tilde {x}$), control inputs ($\tilde {u}$), and output signals (y) are defined as follows:

40 $${\tilde {x}^T}=\left[ {\begin{array}{*{20}{c}} {{{\tilde {i}}_{Lm}}}&{{{\tilde {v}}_{C1}}}&{{{\tilde {v}}_{C2}}}&{{{\tilde {v}}_{C3}}}&{{{\tilde {v}}_{CO}}} \end{array}} \right]$$

41 $$\tilde {u}=\left[ {\tilde {d}} \right]$$

42 $${y^T}=\left[ {{I_{Lm}}} \right]$$

The pole placement technique allows for the positioning of the poles in a closed-loop system at desired locations, provided that the system is completely controllable regarding its state variables. The controllability matrix for the proposed circuit is formulated as follows:

43 $${\Phi _C}=\left[ {B \vdots AB \vdots {A^2}B \vdots \cdots \vdots {A^{n - 1}}B} \right]$$

The system is fully controllable when the rank of ${\Phi _C}$ is equal to 5, matching the number of state variables$\tilde {x}$. In conclusion, the subsequent two integral states are identified as follows:

44 $$\dot {q}(t)=r(t) - y(t)=r(t) - {\tilde {i}_{Lm}}(t)$$

By incorporating these newly identified integral states, the state and output equations are redefined as follows:

45 $$\begin{gathered} \left[ {\begin{array}{*{20}{c}} {\dot {\tilde {x}}(t)} \\ \cdots \\ {\dot {q}(t)} \end{array}} \right]=\left[ {\begin{array}{*{20}{c}} A& \vdots &0 \\ \cdots & \vdots & \cdots \\ { - C}& \vdots &0 \end{array}} \right]\left[ {\begin{array}{*{20}{c}} {\tilde {x}(t)} \\ \cdots \\ {q(t)} \end{array}} \right]+\left[ {\begin{array}{*{20}{c}} B \\ \cdots \\ 0 \end{array}} \right]\tilde {u}(t)+\left[ {\begin{array}{*{20}{c}} 0 \\ \cdots \\ I \end{array}} \right]r(t) \hfill \\ y(t)=\left[ {\begin{array}{*{20}{c}} C& \vdots &0 \end{array}} \right]\left[ {\begin{array}{*{20}{c}} {\tilde {x}(t)} \\ \cdots \\ {q(t)} \end{array}} \right] \hfill \\ \end{gathered}$$

In the equation above, r(t) represents the input reference vector, which is formulated as:

46 $$r(t)={\left[ {{I_{Lm,ref}}} \right]^T}$$

As derived from equation (90), the new matrixes $\bar {A}$ and $\bar {B}$ are investigated as follows:

47 $$\bar {A}=\left[ {\begin{array}{*{20}{c}} A& \vdots &0 \\ \cdots & \vdots & \cdots \\ { - C}& \vdots &0 \end{array}} \right],\bar {B}=\left[ {\begin{array}{*{20}{c}} B \\ \cdots \\ 0 \end{array}} \right]$$

The controllability matrix for the system outlined in equation (90) is defined as follows:

48 $${\bar {\Phi }_C}=\left[ {\begin{array}{*{20}{c}} B& \vdots &{A{\Phi _C}} \\ \cdots & \vdots & \cdots \\ 0& \vdots &{ - C{\Phi _C}} \end{array}} \right]=\underbrace {{\left[ {\begin{array}{*{20}{c}} B& \vdots &A \\ \cdots & \vdots & \cdots \\ 0& \vdots &{ - C} \end{array}} \right]}}_{M}\left[ {\begin{array}{*{20}{c}} I& \vdots &0 \\ \cdots & \vdots & \cdots \\ 0& \vdots &{{\Phi _C}} \end{array}} \right]$$

If ${\Phi _C}$ is of full rank, then the system the system represented by equation (90) is considered fully controllable when the rank of matrix M reaches n + m (where n denotes the count of state variables and m signifies the number of output signals). Consequently, the matrix K can be derived in the following manner:

49 $$\tilde {u}(t)= - K\left[ {\begin{array}{*{20}{c}} {\tilde {x}(t)} \\ \cdots \\ {q(t)} \end{array}} \right]= - \left[ {\begin{array}{*{20}{c}} {{K_x}}& \vdots &{{K_q}} \end{array}} \right]\left[ {\begin{array}{*{20}{c}} {\tilde {x}(t)} \\ \cdots \\ {q(t)} \end{array}} \right]$$

Where K_x_ and K_q_ are as follows:

50 $$\begin{gathered} {K_x}=\left[ {\begin{array}{*{20}{c}} {{K_{11}}}&{{K_{12}}}&{{K_{13}}}&{{K_{14}}}&{{K_{15}}} \end{array}} \right] \hfill \\ {K_q}=\left[ {{{K^{\prime}}_{11}}} \right] \hfill \\ \end{gathered}$$

By substituting equation (94) into equation (90), the resulting expression is obtained as follows:

51 $$\begin{gathered} \left[ {\begin{array}{*{20}{c}} {\dot {\tilde {x}}(t)} \\ \cdots \\ {\dot {q}(t)} \end{array}} \right]=\left[ {\begin{array}{*{20}{c}} {A - B{K_x}}& \vdots &{ - B{K_q}} \\ \cdots & \vdots & \cdots \\ { - C}& \vdots &0 \end{array}} \right]\left[ {\begin{array}{*{20}{c}} {\tilde {x}(t)} \\ \cdots \\ {q(t)} \end{array}} \right]+\left[ {\begin{array}{*{20}{c}} 0 \\ \cdots \\ I \end{array}} \right]r(t) \hfill \\ y(t)=\left[ {\begin{array}{*{20}{c}} C& \vdots &0 \end{array}} \right]\left[ {\begin{array}{*{20}{c}} {\tilde {x}(t)} \\ \cdots \\ {q(t)} \end{array}} \right] \hfill \\ \end{gathered}$$

**Fig. 9:**
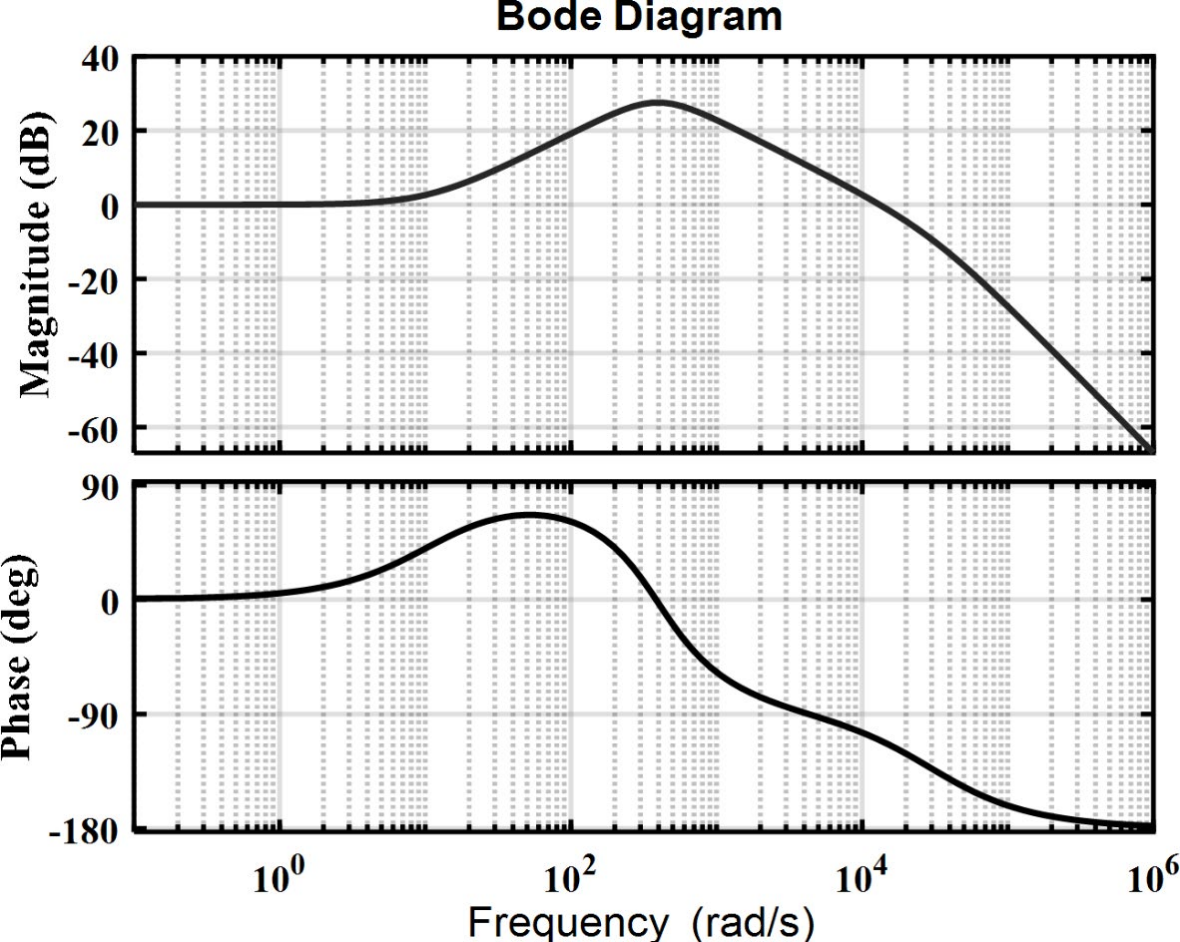
Bode analysis for the transfer function of the coupled inductor current.

To achieve the desired Gain Margin (GM ≥ 10) and Phase Margin (60 ≤ PM ≤ 80), an iterative trial-and-error method is employed to adjust the positions of the closed-loop poles. This process is visualized in the Bode plot of the converter’s control system, as shown in Fig. 9. The plot reveals that the gain margin for the inductor Lm surpasses 10 (GM(i_Lm_) > 10), while the phase margin for the closed-loop path of i_Lm_ is 69.5116, which is considered acceptable.

## Comparison study

**Fig. 10:**
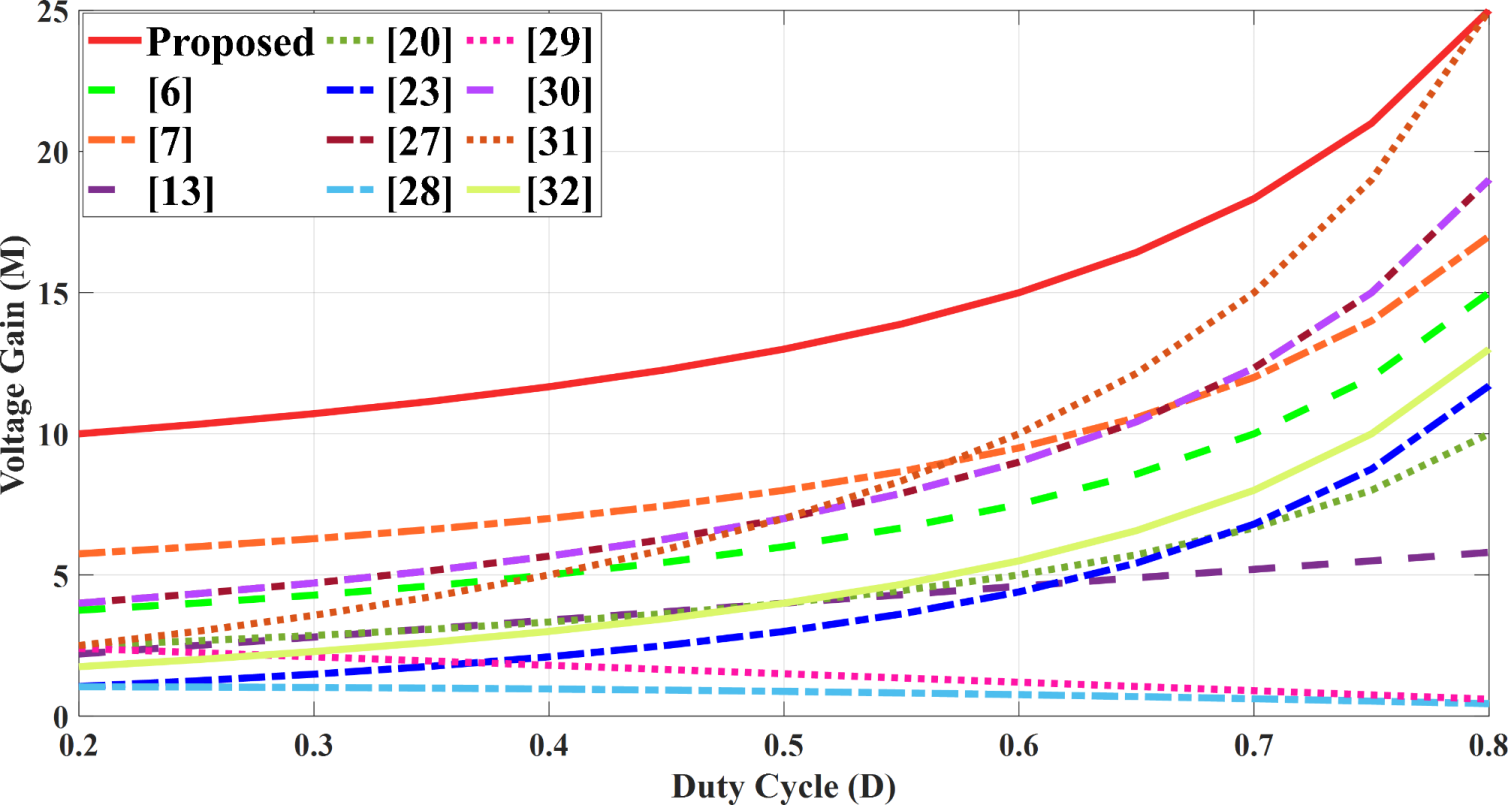
Voltage gain changes across various duty cycles for the evaluated step-up circuits.

**Fig. 11:**
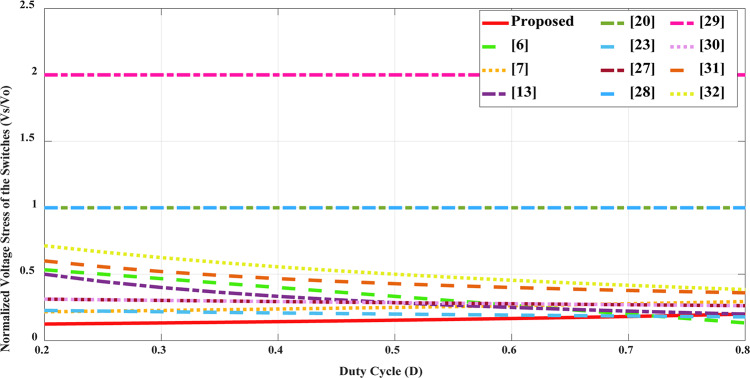
Comparison of the maximum voltage stress on switches versus duty cycle.

**Fig. 12:**
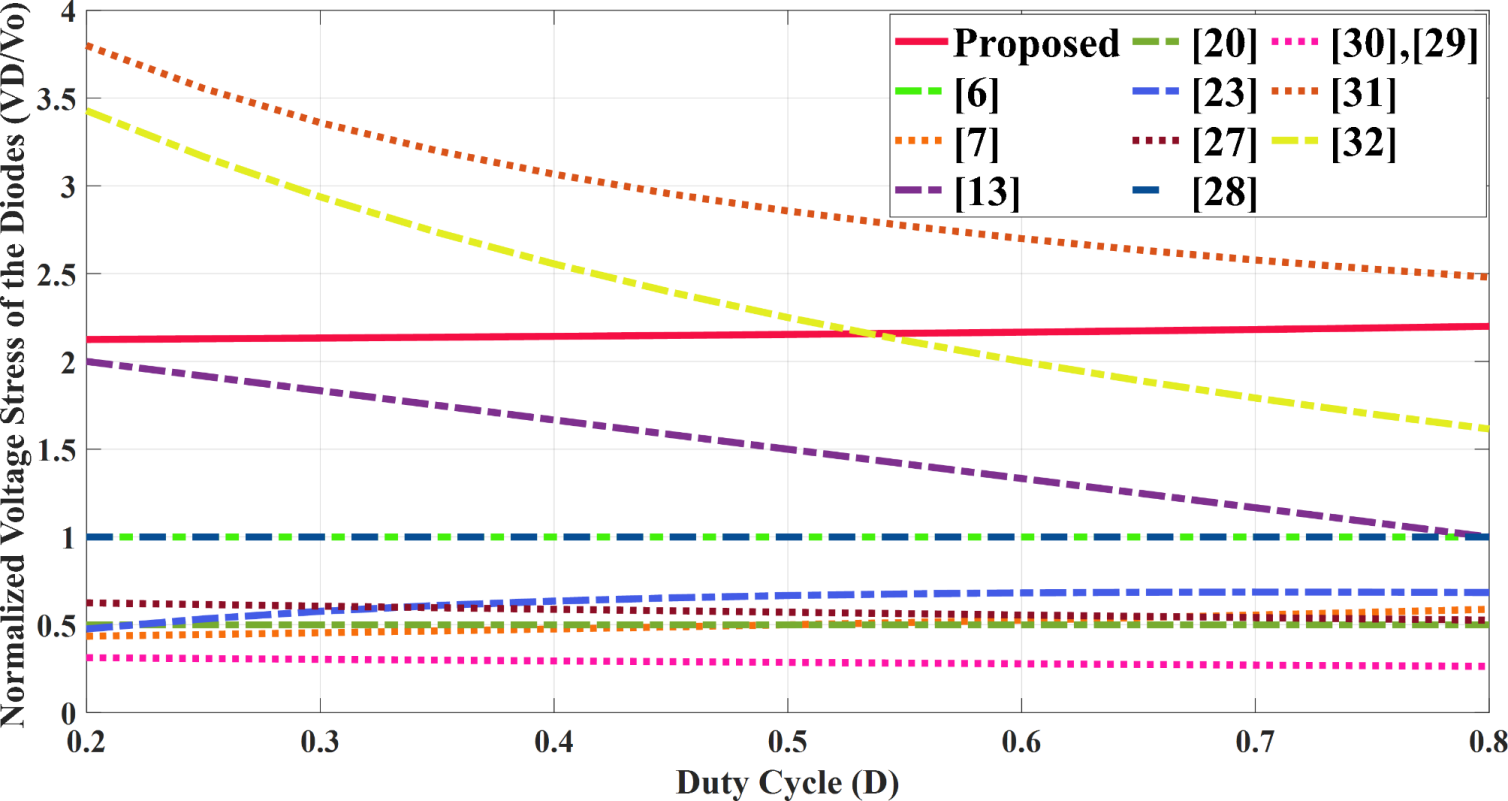
Comparison of the maximum voltage stress on diodes versus duty cycle.

To substantiate the merits of the proposed structure and performance, it was rigorously compared with several existing references. Table 2 highlights key aspects of the suggested topology compared to other designs, focusing on factors such as voltage gain, normalized voltage stress on power switches and diodes, component count, and additional characteristics like efficiency, switching frequency, output power, and whether there is a common ground between the input source and output load. The second column of the table presents a breakdown of the number of switches, diodes, capacitors, and inductors used in each configuration. The overall component count in the introduced topology is comparable to or lower than that of most high step- up converters, including those referenced in^13,20,23,27,28,30–32^. Evaluating the total number of components alone is insufficient for a comprehensive assessment. Additionally, Fig. 10 presents a comparison of voltage gains across different duty cycles for several converter designs, demonstrating that the proposed topology delivers a higher voltage gain at *n* = 2 compared to other configurations under the same duty cycle. Figure 11 displays the relationship between normalized voltage stress on the power switch and the duty cycle, while Fig. 12 illustrates how the normalized voltage stress on the diodes varies with the duty cycle. Considering Fig. 11 and the six column of Table 2, that the voltage stress across the power switch is lower in the proposed converter compared to other topologies. In a similar vein, when comparing voltage stress on the output diode is primary considered, as the most stress in this type of structure. Across the entire range (0 to 1), the voltage stress on the output diode in the proposed topology is lower than structures in references^31,32^. Furthermore, the suggested topology is common grounded structure. As shown in Table 2 there is no common ground in the configurations presented in^13,27,30,32^. As a result of these advantages, the proposed converter can operate at a higher power levels than other converters, while maintaining suitable efficiency, compactness, and overall cost- effectiveness. The efficiencies of the presented topology and other references at different switching frequencies and output power levels are provided. Notably, the efficiency in^29^ is higher than the suggested topology, achieving 96.4% at a 50 kHz switching frequency and 200 W of output power. In comparison, the converter introduced in this paper achieves an efficiency of 95.8% under the same switching frequency and 400 W output power. Table 3 presents a cost comparison between the proposed converter and other designs. It reveals that the proposed converter is the most economical option. The projected component costs were gathered from listings on platforms such as AMAZON, MOUSER, and EBAY, and are detailed in Table 3.

**Table 2:** Comparison between the proposed converter and other structures.

Ref.	Voltage Gain	Number of components				$\eta (\% )$	$f_{s} \,(KHz)$	Maximum normalized voltage stress on the semiconductors		Vin/VoPo(W)	C.G^*^
		S	D	C	L			$${\raise0.7ex\hbox{${V_{s} }$} \!\mathord{\left/ {\vphantom {{V_{s} } {V_{o} }}}\right.\kern-\nulldelimiterspace} \!\lower0.7ex\hbox{${V_{o} }$}}$$	$${\raise0.7ex\hbox{${V_{D} }$} \!\mathord{\left/ {\vphantom {{V_{D} } {V_{o} }}}\right.\kern-\nulldelimiterspace} \!\lower0.7ex\hbox{${V_{o} }$}}$$		
^6^	$\frac{{N + 1}}{{1 - D}}$	1	2	1	2	97	100 kHz	$\frac{{NV_{o} (1 - D)}}{{1 + N}}$	${V_{o} }$	40/400 250 W	✔
^7^	$\frac{{2N + 1 - ND}}{{1 - D}}$	1	4	4	1	95.14	50 kHz	$\frac{1}{{2N + 1 - ND}}$	$\frac{N}{{2N + 1 - ND}}$	20/260 200 W	✔
^13^	$\frac{{(3N - 1)D + N - 1}}{{(N - 1)(1 - D)}}$	3	4	3	2	95	25 kHz	$\frac{{1 + D_{1} }}{{3 + D_{1} - D_{2} }}$	$\frac{{7 - D_{1} - 4D_{2} }}{{3 + D_{1} - D_{2} }}$	20/400 300 W	✘
^17^	$\frac{{N_{D} + 1}}{{1 - D}}$	2	2	1	2	91	50 kHz	N.R	N.R	24/100 300 W	✔
^20^	$\frac{2}{{1 - D}}$	1	6	6	1	95	50 kHz	$\frac{{1 + D(k - 1)}}{2}$	$\frac{{V_{o} }}{2}$	20/200 200 W	✔
^23^	$\frac{{1 + D + ND(1 + D)}}{{2(1 - D)}}$	1	5	4	1	95.15	15 kHz	$\frac{{V_{o} }}{{N + ND + 2}}$	$\frac{{2NDk(1 - D)}}{{(1 + D) + ND(1 + D)(1 - D)}}$	20/200 150 W	✔
^27^	$\frac{{3 + D}}{{1 - D}}$	6	14	8	6	97.2	25 kHz	$\frac{1}{{3 + D}}$	$\frac{2}{{3 + D}}$	24/207 484 W	✘
^28^	$\frac{{3D + N}}{{N(1 - D)}}$	4	1	4	2	97	100 kHz	$V_{o}$	$V_{o}$	48/120 100 W	✔
^29^	$\frac{{2N - 1}}{{(N - 1)(1 - D)}}$	4	0	3	1	96.4	50 kHz	$2V_{o}$	$\frac{{NV_{o} }}{{2N - 1}}$	25/200 200 W	✔
^30^	$\frac{{1 + N_{3} + D}}{{1 - D}}$	2	4	4	1	95	50 kHz	$\frac{1}{{1 + N_{3} + D}}$	$\frac{{N_{3} }}{{1 + N_{3} + D}}$	50/400 500 W	✘
^31^	$\frac{{4 + 5D}}{{1 - D}}$	3	12	1	6	95.6	20 kHz	$\frac{{1 + D}}{{1 + 5D}}$	$\frac{{6 + 8D}}{{1 + 5D}}$	20/160 200 W	✔
^32^	$\frac{{1 + 3D_{1} - D_{2} }}{{1 - D_{1} - D_{2} }}$	3	8	1	4	92.04	20 kHz	$\frac{{1 + D_{1} - D_{2} }}{{1 - 3D_{1} - D_{2} }}$	$\frac{{5 - D_{1} }}{{1 + 3D_{1} - D_{2} }}$	3/78 40 W	✘
PC	$\frac{{3kn - 2Dkn - D + 3}}{{1 - D}}$	1	4	4	1	95.8%	50 kHz	$\frac{1}{{3kn - 2Dkn - D + 3}}$	$\frac{{kn + 1}}{{3kn - 2Dkn - D + 3}}$	20/260 400 W	✔

PC*=Proposed Converter *Common Ground

**Table 3:** Cost comparison between the suggested topology and other converters

Ref	Cost of switches	Cost of diodes	Cost of capacitors	Cost of cores	Total Cost
PC^*^	1 × 0.363$	3 × 0.476$ 1 × 0.379$	3 × 0.51$ 1 × 0.43$	1 × 2.8$	6.931$
^7^	1 × 1.2$	4 × 0.7$	3×0.1.68$ 1×0.4.39$	1 × 3.2$	16.63$
^13^	2 × 2$	1 × 0.59$ 1 × 0.7$	3×0.055$	1 × 2.8$	9.74$
^20^	1 × 1.2$	4 × 0.45$ 2 × 0.7$	5 × 2.78$ 1 × 4.39$	2 × 3.2$	25.89$
^28^	4 × 1.22$	4 × 2$	2 × 2.18$ 1 × 11.99$	1 × 2.2$	20.77$

PC*=Proposed Converter

Table 4 presents a comprehensive analysis of the proposed converters volume and size distribution. It becomes clear that the coupled inductor accounts for the largest portion of the overall volume, closely followed by the capacitors, with the high-voltage side capacitor being the primary contributor. In contrast, the semiconductor components have a much smaller impact on the total volume. Furthermore, Table 5 compares power density, revealing that the proposed converter achieves a theoretical power density of 400 watts within a volume of 37,694.48 cubic millimeters. This comparison underscores the advantages of the proposed design, such as its superior voltage gain and minimized voltage stress on both the power switches and diodes. Moreover, the reduced voltage stress on the power switch enables the use of a smaller inductor within the circuit.

**Table 4:** Power density of the suggested topology.

Components	Specification	Volume
Switches	IRF2807PbF Length:23.74 mm Width:10.67 mm Thickness:4.83 mm	2984.84mm^3^
Diodes	BYV32E-200 Length:31 mm Width:10.3 mm Thickness:4.7 mm SBR10U300CT Length:29.15 mm Width:10.31 mm Thickness:4.67 mm	(3 × 1500.71)+(1403.5)= 5905.63mm^3^
Capacitors	C_1_,C_2_,C_3_ Diameter:13 mm Height:20 mm Co Diameter:15 Height:30 Diameter:16 Height:25	(3 × 2654.65)+(5301.44)= 13265.39mm3
Coupled Inductor	EE42/21/15 core	17300mm^3^
	**Total volume**:	37694.48mm^3^
	**Power Density**:	10.61mW/mm^3^

**Table 5:** A comparison of power density between the proposed converter and other existing converters.

Ref	S.V* (mm3)	D.V* (mm3)	C.V* (mm3)	L.V* (mm3)	T.V* (mm3)	*P*.D* (mm3)
Prop	1223.46	5905.63	13265.39	17,300	37694.48	400 W/37694.48= 10.61 mW/mm3
^7^	2984.84	5845.48	53053.65	43,900	105783.28	200 W/105783.28= 1.89 mW/mm3
^13^	6194.4	3073.94	2213.07	17,300	28781.41	200 W/28781.41= 6.948 mW/mm3
^20^	2984.84	6079.7	50681.74	43,900	103646.28	200 W/103646.28= 1.93 mW/mm3
^28^	3243.55	1350	1326.26	701	6620.81	100 W/6620.81= 15.10 mW/mm3

^*S.V: Switches Volume. *D.V: Diodes volume. *C.V: Capacitors Volume^.

^*I.V: Inductors Volume. *T.V: Total Volume. *P.D: Power Density^.

## Experimental results

**Fig. 13:**
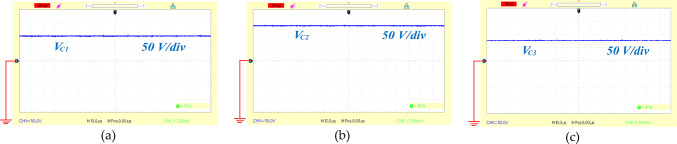
The experimental voltage waveforms of capacitors, (**a**) voltage across the capacitors C_1_, (**b**) voltage across the capacitor C_2_, (**c**) voltage across the capacitors C_3_.

**Fig. 14:**
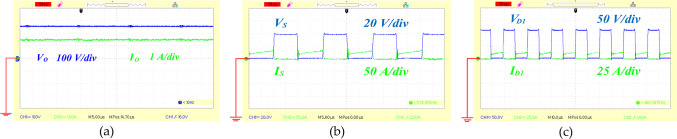
The experimental waveforms of output port, power switch, and diode D_1_, (**a**) voltage and current of the output port, (**b**) voltage and current of the power switch, (**c**) voltage and current of the diode D_1_.

**Fig. 15:**
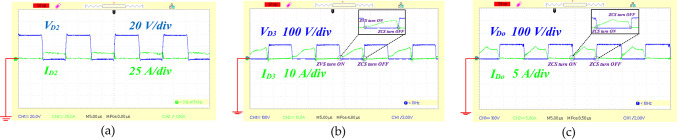
The experimental waveforms of diodes, (**a**) voltage and current of the diode D_2_, (**b**) voltage and current of the diode D_3_, (**c**) voltage and current of the diode D_O_.

To affirm the theoretical findings and practical application of the proposed circuit, a 400 W experimental prototype was created. The key specifications of this high step-up design are detailed in Table 6. Figure 13(a), 13(b) and 13(c) illustrate the voltages across the capacitors C_1_, C_2_ and C_3_ which were measured at 97, 138, and 78 volts respectively. According to Eqs. (13), (14), and (15) the expected capacitor voltages are 100 V for C_1_, 140 V for C_2_, and 80 V for capacitor C_3_. Figure 14(a), shows the recorded output voltage and current measured at 255 V and 1.5 A, respectively. The predicted output voltage from Eq. (16) is about 260 V, which closely aligns with the experimental data, confirming the proper functionality of the proposed topology. The voltage across the single power switch was recorded at 39 V, as depicted in Fig. 14(b), with the switch carrying a current of 40 A. In Fig. 14(c), the voltage and current for diode D_1_ are shown to be 200 V and 5 A, respectively. Additionally, 15(a) illustrates both the voltage and current for diode D_2_, and measured 40 V and 13 A respectively. Figure 15(b) depicts diode D_3_ operating under ZVS and ZCS conditions, with measurements of 120 V and 9 A. Similarly, diode D_o_ shows ZCS behavior, with 120 V and 4 A recorded in Fig. 15(c). Collectively, Figs. 13 and 14, and 15 indicate that the experimental results of the proposed converter align closely with the theoretical projections, thereby confirming its effective performance.

**Table 6:** Component characteristic of the implemented circuit.

Rated power (*P*_Out_)	400 W
Input voltage	20 V
Output voltage	260 V
Switching Frequency (*f*_*s*_)	50 kHz
Turns Ratio *n*_*1*_ (*N*_*S*_*/N*_*P*_), *n*_*2*_ (*N*_*t*_*/N*_*P*_)	2, 2
Magnetizing Inductor (*L*_*m*_)	500 *µH*
Leakage Inductor (*L*_*k*_)	3 *µH*
Power Switch	IRF2807PbF ${r_S}=$ 13*m*Ω
Diode *D*_*1*_	SBR10u300CT ${r_{D1}}=$ 6*m*Ω
Diodes (*D*_*2*_, *D*_*3*_,*D*_*O*_)	BYV32E-200 ${r_D}=$ 6*m*Ω
Capacitors (*C*_*1*_, *C*_*2*_,*C*_*3*_)	47 µF/200V, ${r_C}=$ 10*m*Ω
Capacitor (*C*_*o*_)	47 µF/350V ${r_{Co}}=$ 10*m*Ω

## Conclusions

This paper introduces a high step-up, non-isolated DC-DC converter that utilizes a single switch alongside a three-winding coupled inductor. This innovative design significantly enhances voltage gain while offering improved efficiency and soft-switching capability for diodes D_3_ and D_O_. It also lowers the voltage stress experienced by semiconductor components and features two adjustable control parameters: the duty cycle and the coupled inductor’s turn ratio. Moreover, the incorporation of a single power switch streamlines the control process of the converter. This configuration enables the achievement of substantial voltage gains without being limited by the duty cycle, allowing for the production of high output voltages with a low duty cycle, thus minimizing conduction losses in the switch.

## Data Availability

All data generated or analyzed during this study are included in this published article.
